# Automated Whole-Body Bone Lesion Detection for Multiple Myeloma on ^68^Ga-Pentixafor PET/CT Imaging Using Deep Learning Methods

**DOI:** 10.1155/2018/2391925

**Published:** 2018-01-08

**Authors:** Lina Xu, Giles Tetteh, Jana Lipkova, Yu Zhao, Hongwei Li, Patrick Christ, Marie Piraud, Andreas Buck, Kuangyu Shi, Bjoern H. Menze

**Affiliations:** ^1^Department of Informatics, Technische Universität München, Munich, Germany; ^2^Department of Nuclear Medicine, Klinikum Rechts der Isar, TU München, Munich, Germany; ^3^Institute of Medical Engineering, Technische Universität München, Munich, Germany; ^4^Department of Nuclear Medicine, Universität Würzburg, Würzburg, Germany

## Abstract

The identification of bone lesions is crucial in the diagnostic assessment of multiple myeloma (MM). ^68^Ga-Pentixafor PET/CT can capture the abnormal molecular expression of CXCR-4 in addition to anatomical changes. However, whole-body detection of dozens of lesions on hybrid imaging is tedious and error prone. It is even more difficult to identify lesions with a large heterogeneity. This study employed deep learning methods to automatically combine characteristics of PET and CT for whole-body MM bone lesion detection in a 3D manner. Two convolutional neural networks (CNNs), V-Net and W-Net, were adopted to segment and detect the lesions. The feasibility of deep learning for lesion detection on ^68^Ga-Pentixafor PET/CT was first verified on digital phantoms generated using realistic PET simulation methods. Then the proposed methods were evaluated on real ^68^Ga-Pentixafor PET/CT scans of MM patients. The preliminary results showed that deep learning method can leverage multimodal information for spatial feature representation, and W-Net obtained the best result for segmentation and lesion detection. It also outperformed traditional machine learning methods such as random forest classifier (RF), *k*-Nearest Neighbors (*k*-NN), and support vector machine (SVM). The proof-of-concept study encourages further development of deep learning approach for MM lesion detection in population study.

## 1. Introduction

Multiple myeloma (MM) is a malignancy accounting for 13% of the hematological cases [1–3]. A characteristic hallmark of MM is the proliferation of malignant plasma cells in the bone marrow [4]. Common symptoms of MM are summarized as CRAB: C for hypercalcemia, R for renal failure, A for anemia, and B for bone lesions. Modern treatments have achieved a 5-year survival rate of 45% [5]. Nevertheless, MM remains an incurable disease at the moment and it usually relapses after a period of remission under therapy. The identification of bone lesions plays an important role in the diagnostic and therapeutic assessment of MM.

Radiographic skeletal survey (whole-body X-ray) is traditionally applied in the characterization of bone lesions of MM. However, it can only display lesions when the trabecular bone has already lost more than 30% around the focal, usually leading to underestimation of lesion extent [6]. 3D computed tomography (CT) allows the detection of smaller bone lesions that are not detectable by conventional radiography [7]. Magnetic resonance imaging (MRI) is also more sensitive than skeletal survey in the detection of MM lesions and it can detect diffuse bone marrow infiltration with good soft tissue differentiation [8, 9]. Comparable high sensitivity in the detection of small bone lesions can be achieved using PET/CT by combining metabolic (^18^F-FDG PET) and anatomical (CT) information [10–13]. The lesions are usually visualized more clearly with the guidance of hotspots in fused images, which can potentially improve the diagnosis and prognosis of MM [14]. Recently, the overexpression of chemokine (C-X-C motif) receptor 4 (CXCR4) has been verified in a variety of cancers, leading to the development of targeted PET tracer such as ^68^Ga-Pentixafor [15]. This emerging tracer has already demonstrated a higher sensitivity in the visualization of MM lesions [16, 17].

Even with advanced imaging, challenges remain in the identification of MM bone lesions. It is commonly seen that dozens of lesions spread across the whole body. Manual reading of all these distributed lesions is usually tedious for physicians and can result in large interobserver variations [18] and may be prone to errors. Although metabolic lesion volume is a prognostic index for the interpretation of MM PET images [19], it is necessary to identify the lesions before the calculation of characteristic quantities such as the maximum of standardized uptake value within tumor (SUVmax) and total lesion evaluation (TLE) [20, 21].

Computer-aided detection (CAD) has been developed to assist radiologists to resolve the critical information from complex data, which improves the accuracy and robustness of diagnosis [22–25]. Machine learning is the engine for typical CAD approaches. Several methods have been developed for lesion detection or tumor screening in oncological applications [26, 27], in which lesion and nonlesion parts are differentiated and segmented. For hybrid imaging, either is characterized with low spatial resolution in PET or features with low contrast in CT, and a direct detection or cosegmentation of tumors in both modalities is difficult. Based on such concern, in [28, 29] a fuzzy locally adaptive Bayesian algorithm has been developed for volume determination for PET imaging and later applied in lung tumor delineation. In [30–33] Markov Random Field (MRF) and graph-cut based methods were integrated to encode shape and context priors into the model. Simultaneously model delineation of regions using random walk and object/background seed localization method were also being employed in joint segmentation. In [34] modality-specific visibility weighting scheme based on a fuzzy connectedness (FC) image segmentation algorithm was proposed to determine the boundary correspondences of lesions in varied imaging modalities.

**Figure 1:**
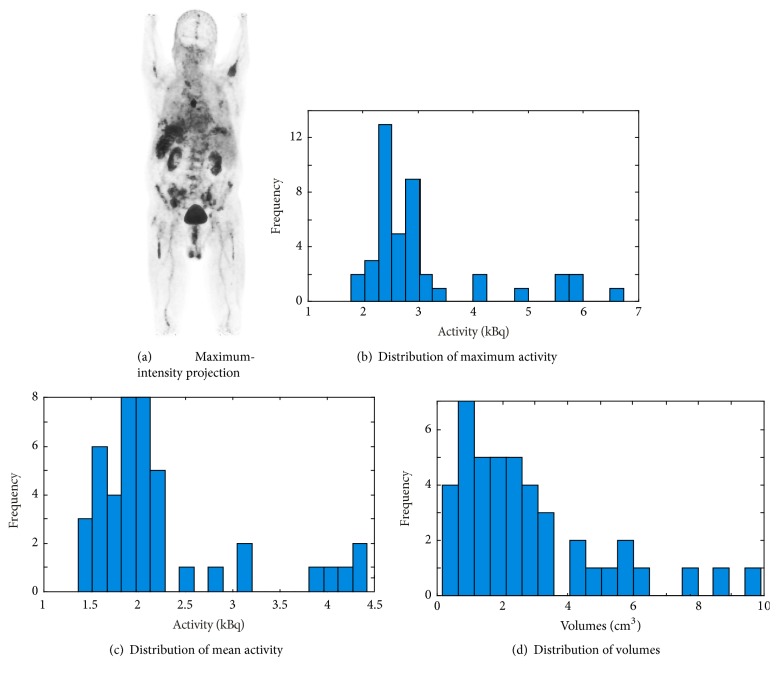
Properties of MM lesions of an exemplary patient with ^68^Ga-Pentixafor PET imaging: (a) maximum-intensity projection of ^68^Ga-Pentixafor PET; (b) histogram distribution of maximum activity of the lesions; (c) histogram distribution of mean activity of the lesions; (d) histogram distribution of volumes of the lesions.

In order to tackle the multiple myeloma lesion detection, classification, or other pathological analysis issues for the bone, several methods have been developed. A virtual calcium subtraction method [35] has been adopted to evaluate MM in the spine. The study of [36] focuses on detecting lesions in femur cortical bones, in which a probabilistic, spatially dependent density model has been developed to automatically identify bone marrow infiltration in low-dose CT. In [37] a semiautomatic software was developed to perform pixel thresholding based segmentation for the assessment of bone marrow metabolism while automatic quantification of bone marrow myeloma volume was conducted in [38]. A hybrid iterative reconstruction technique was used to compare the diagnostic performance of conventional radiography (CR) and whole-body low-dose computed tomography (WBLDCT) with a comparable radiation dose reconstructed for MM staging [39]. However, none of the above-mentioned approaches can be directly transferred to automatically detect systemic bone lesions on PET imaging. As is shown in Figure 1, the ^68^Ga-Pentixafor PET imaging has a large variation in uptake and size even among the lesions in the same patient. Such heterogeneity in lesion size and tracer uptake in the complex context of various nonspecific overexpression makes the whole-body detection of all the lesions extremely difficult. To the best of our knowledge, no effective CAD methods have been presented for automated detection of MM bone lesions in the full body.

The emergence of deep learning methods largely exceeds human perception power in extracting useful information from large amount of data such as images and conventional machine learning methods in many applications [26, 40, 41]. They have already demonstrated advantages in computerized diagnosis on medical images, such as abnormality detection [42, 43]. Convolutional neural networks (CNNs) are the driving force among many network architectures, and the current state-of-the-art work largely relies on CNN approaches to address the common semantic segmentation or detection tasks [44]. The combination of convolutional and deconvolutional layers can well extract high-level contextual and spatial features hierarchically. CNN architecture such as U-Net offers a 2D framework to segment biomedical images by using a contracting path for contextual extraction and a symmetric reversed path for object localization [45]. U-Net has been extended to a 3D version as V-Net [46] and achieves promising results by introducing an optimized objective function to train the model end-to-end. Similar 3D CNNs have been presented in [47] to learn intermediate features for brain lesion segmentation. A cascaded CNN has been developed to first segment the liver and then the liver lesions in [48].

This paper explores the advantages of cascaded CNNs in lesion prediction and segmentation with the aim of detecting MM bone lesion in a full body manner. For the first time, deep learning method is developed to combine anatomical and molecular information of ^68^Ga-Pentixafor PET/CT imaging to support whole-body lesion identification. Besides employing V-Net, two enhanced V-Nets are cascaded to build a W-shaped framework to learn the volumetric feature representation of the skeleton and its lesions from coarse to fine. The whole network requires only minimal preprocessing and no postprocessing. We testify the algorithm on 70 digital phantoms generated by realistic simulation of ^68^Ga-Pentixafor PET images to demonstrate the applicability of deep learning architectures in hierarchically extracting features and predicting the MM bone lesions. For proof-of-concept, the methods were further evaluated on 12 clinical PET/CT data and the results demonstrate the potential to improve MM bone lesion detection. In addition, we compared the proposed approach with several traditional machine learning methods, including random forest classifier, *k*-Nearest Neighbor (*k*-NN) classifier, and support vector machine (SVM) algorithm, in which cases the advantages of deep learning methods are more evidently shown.

## 2. Methods and Materials

### 2.1. Deep Learning Methods: V-Net and W-Net

In this study, we investigate a widely used CNN-based deep learning architecture, V-Net, for 3D volumetric image segmentation [46] on CT and PET images. 3D convolutions are performed aiming to extract features from both modalities. At the end of each stage, to reduce its resolution by using appropriate stride, the left part of the V-Net consists of a compression path, while the right part decompresses the signal until its original size is reached. Convolutions are all applied with appropriate padding. We assembled PET and CT into two channels of combined images for lesion segmentation.

**Figure 2:**
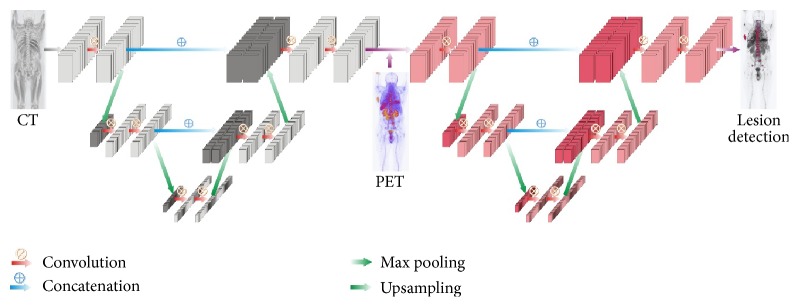
Overview of a simplified W-Net architecture.

In particular, we cascaded two V-Nets to form a W-Net architecture to improve the segmentation to bone-specific lesions in this study. As illustrated in Figure 2, there is a compression downward path, followed by an approximately symmetric decompression path inside each V-Net. The former cuts the volumetric size and broadens the receptive field along the layers, while the latter functions the opposite way to expand the spatial support of the lower resolution feature maps. For both contracting and expanding paths, we use the 3 × 3 × 3 kernel for convolution and a stride of two for max pooling or upsampling. For the first V-Net, only volumetric CT data is fed into the network in order to learn anatomical knowledge about the bone. The outcome builds a binary mask for the skeleton, which adaptively offers geometrical boundary for lesion localization. The second V-Net then adds both PET/CT and the output from the first network as the total input, of which PET/CT provides additional feature information to jointly predict the lesion. Since lesions have comparatively smaller size than the bone, the deeper a network goes, the more detailed information will vanish even if adding concatenations from layers in the contracting path. For the W-Net, we use five layers in the first V-Net and three layers for the second V-Net. For the single V-Net architecture, 3 layers are adopted.

All the experiments are implemented on Theano using Python 2.7 and all the PET/CT volumes are trained on NVIDIA TITAN X with a GPU memory of 12 GB. We employed 3-fold cross validation to test the prediction accuracy.

There exists high imbalance in the dataset, especially for lesion labels with very small sizes. In order to better track tiny lesions and balance different sizes of the bone systemically, we also adopt a similar weight balance strategy as in [48]. For the input CT volume *V* and a given voxel *i*, there is a set of labels containing two values, of which 0 denotes nonbone regions and 1 denotes bones. As for the PET volumes in the binary label set, 0 denotes nonmyeloma part and 1 indicates MM lesions. We define *p*(*x*_*i*_ = *l*∨*V*) to be the probability that a voxel *i* is being assigned a label *l* given the volume *V*, with the label set *l* ∈ [0,1]. Then a cross-entropy loss function *L* is defined as follows:

(1) $$\begin{array}{c} L=\frac{-1}{N}\overset{N}{\underset{i=1}{\sum }}\omega_{i}^{label}\left[\;{\hat{p}}_{i}\log p_{i}+\left(\;1-{\hat{p}}_{i}\;\right)\log\left(\;1-p_{i}\;\right)\;\right]. \end{array}$$

In (1), ${\hat{p}}_{i}$ is the ground truth while *p*_*i*_ is the probability assigned to voxel *i* when it belongs to the foreground. *ω*_*i*_^label^ is set to be inversely proportional to the number of voxels that belong to a certain label.

Besides, we adopt another balance implementation, which is patch-based balance strategy. We subsample the training dataset to bridge the gap between the labels (bone and lesion) and the background. For each patient, we pretrain our network by first extracting patches of size (64 × 64 × 64) across the whole patient volume, with an overlap of 5 voxels in all directions. We then select the top 30 patches per patient volume based on the ratio of label to background in each patch. These selected patches improve the total label to background ratio for the bone label from a percentage of 12.35% to 20.16% and for the lesion label from a percentage of 2.54% to 7.01%.

With the class balancing loss function and the patches used in V-Net, we pretrain the network for 1000 iterations. Stochastic gradient descent with a learning rate of 0.001 and a momentum of 0.95 is performed for every 100 iterations. We then use the entire training set to fine-tune the network until it converges following the same setup as is adopted in the pretraining process. This training scheme is employed for both bone and lesion segmentation tasks.

Dice score was calculated to estimate the segmentation accuracy. In addition, the lesionwise detection accuracies (sensitivity, specification, and precision) were summarized on the segmented results based on the criteria of patch overlapping. Patches of size 9 × 9 × 9 were generated across the lesions with an overlap of 4 voxels being added in all three directions. A lesion was considered as detected when the amount of lesion labels that fell into the patch was above 10%.

We calculate precision and other relevant metrics via patch overlapping, where the annotated ground truth patch is considered positive if at least 10% of the total voxels in the patch are a lesion and negative otherwise.

### 2.2. Test on Realistic PET Phantoms

To evaluate the performance of deep learning methods for lesion detection, we generated realistic digital phantoms of ^68^Ga-Pentixafor PET scans. Digital phantoms using physically based simulations provide ground truth to in-depth evaluate the performance of algorithms [49]. Realistic PET activities extracted from patient data were assigned to a whole-body phantom with CT and anatomical labels such as liver, spleen, and kidney. Bone lesions of various sizes, shapes, and uptakes at different locations were generated randomly in skeleton of phantoms to represent a large diversity of lesion presentations. The CT values were modified accordingly by considering severity of lesions. Realistic PET measurements of the phantoms were simulated by a forward projection model following the procedures described in [50]. This model includes object attenuation as well as the system geometry resembling the characteristics of a real clinical scanner (Siemens Biograph mMR [51]). With the scanner geometry described above, scattered events within the phantom and detectors were simulated using GATE V6. These events were sorted out from the GATE output and formed the expectation of scatter sinogram. In this simulation, 30% scattered and 30% uniformly distributed random events were included considering a large positron range of Ga-68. Poisson noise was then generated in each sinogram bin. Finally, a set of sinograms (90 bins and 160 projections) was generated with the expectation of the total counts to be 1 million. In total, 70 digital phantoms of different lesions were simulated.

### 2.3. Test on Clinical Data

12 patients (3 female and 9 male) with histologically proven primary multiple myeloma disease were referred for ^68^Ga-Pentixafor PET/CT imaging (Siemens Biograph mCT 64; Siemens Medical Solutions, Germany). Approximately 90 to 205 MBq ^68^Ga-Pentixafor was injected intravenously 1 hour before the scan. A low-dose CT (20 mAs, 120 keV) covering the body from the base of skull to the proximal thighs was acquired for attenuation correction. PET emission data were acquired using a 3D mode with a 200 × 200 matrix for 3 min emission time per bed position. PET data were corrected for decay and scattering and iteratively reconstructed with attenuation correction. This study was approved by the corresponding ethics committees. Patients were given written informed consent prior to the investigations.

The coregistration of PET and CT was visually inspected using PMOD (PMOD Technologies Ltd., Switzerland). With the fusion of PET and CT, all the lesions were manually annotated under the supervision of experienced radiologist, then each lesion was segmented by local thresholding at half maximum.

### 2.4. Comparison with Traditional Machine Learning Methods

Traditional machine learning methods [52] including random forest, *k*-NN, and SVM were employed in this study for the comparison with deep learning methods. The patch-based intensity information was extracted as features for different algorithmic implementation. Multimodality features were obtained by taking the PET and CT intensities patchwise with a size of 3 × 3 × 3 in order that neighbor and intensity information can be encoded. For training, a total of 2000 lesion samples (patches) and 2000 nonlesion samples for each data volume were randomly selected and normalized to form the feature space. Each sample in the training/test set was represented as an intensity-based feature vector of 54 dimensions. Then the principal component analysis (PCA) was applied to reduce the dimensionality to 15 and the grid search with 3-fold cross validation was used to select the parameters. For random forest, the number of trees *n* was set to 20. For* k*-NN, the number of neighbors *k* was set to 15. For SVM, we choose a linear kernel, and the penalty parameter of the error term C was set to 0.5.

## 3. Results and Discussions

**Figure 3:**
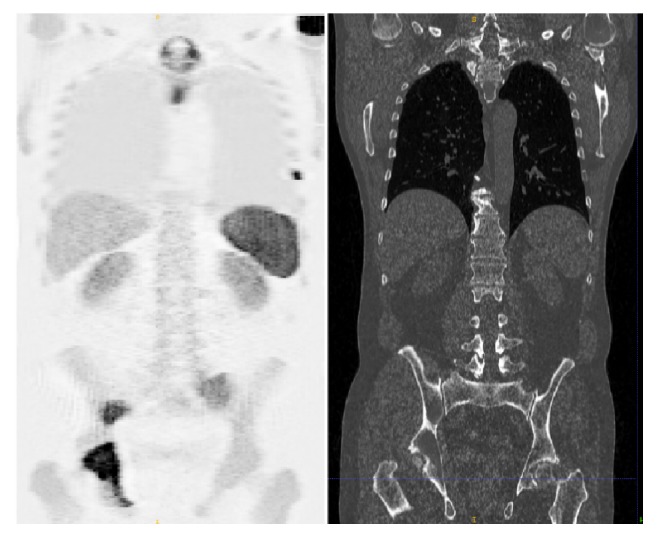
Synthetic PET data generating from digital phantom and its corresponding CT scan.

**Figure 4:**
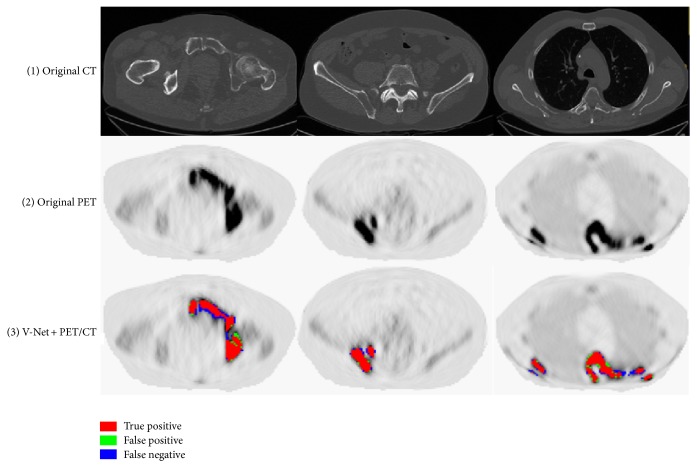
Exemplary detection results of phantom study: (1) the original axial CT slices; (2) the corresponding PET slices; (3) MM lesion prediction using V-Net.

An exemplary coronal slice of simulated ^68^Ga-Pentixafor PET and its corresponding CT scan are shown in Figure 3. The simulated PET images present visual similarities to real PET measures. The detection results of 3 slices of different body regions in axial plane are shown in Figure 4 and the test results on 70 of these realistic digital phantoms are listed in Table 1. It achieves specificity as high as 99.68% and the Dice score is also remarkable with a value of 89.26%, which confirm that deep learning method has the potential to detect the whole-body MM lesions.

The comparisons between V-Nets and W-Net using clinical dataset are summarized in Table 2. For the deep learning methods, the combination of PET and CT for V-Net improves the Dice score (69.49%) compared to V-Net with CT alone (26.37%) or PET alone (28.51%). For lesionwise accuracy, the combination of PET and CT for V-Net achieves higher specificity (99.51%) than V-Net using CT (94.43%) or PET (96.04%) and lower sensitivity than V-Net with CT alone (73.18%). For V-Net, the combination of CT and PET can improve the lesion segmentation; however, it does not bring much benefit to the sensitivity. This is because CT volume represents good anatomical structure and is sensitive to abnormal structure of lesions. The mixture usage of CT and PET in lesion detection does not increase the possibility of capturing such feature.

W-Net, which also combines PET and CT, reaches the highest segmentation accuracy (Dice score 72.98%) and lesion detection accuracy (sensitivity 73.50%, specificity 99.59%, and precision 72.46%). In contrast to V-Net, W-Net distinguishes the information on CT and PET, and the extracted CT skeleton can be utilized as a type of regularization. The maximization of information utilization improves the segmentation and lesion detection. However, given the expensive computation by adding an extra V-Net, the sophisticated W-Net only slightly improves the performance (around 2% to 4%) compared to V-Net with PET/CT input. This can be elucidated in two aspects. On one hand, the hybrid input already contains anatomic information, which is encoded and learnt as important features by the single V-Net. On the other hand, the overall performance of the W-Net may be restricted by the first V-Net. If the skeleton mask is not correctly labeled, its segmentation error will be propagated to the second V-Net and once again cause negative effect on subsequent lesion detection. Further improvement of the individual V-Net may improve the overall performance. Besides, all the methods obtain high specificity (true negative rate) of more than 90%, which demonstrate that the deep learning methods can properly exclude nonlesion parts.

**Figure 5:**
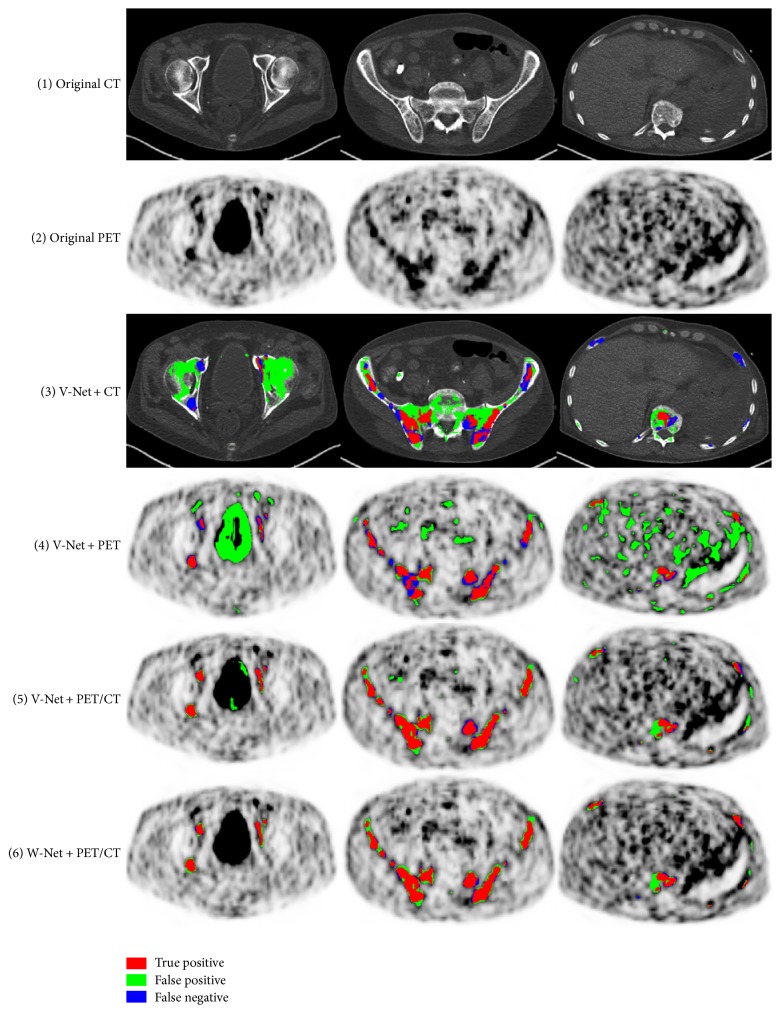
Exemplary detection results of V-Nets and W-Net: (1) the original axial CT slices; (2) the corresponding PET slices; (3) MM lesion prediction using CT alone in V-Net; (4) MM lesion prediction using PET alone in V-Net; (5) MM lesion prediction using PET/CT in V-Net; (6) MM lesion detection using W-Net.

**Figure 6:**
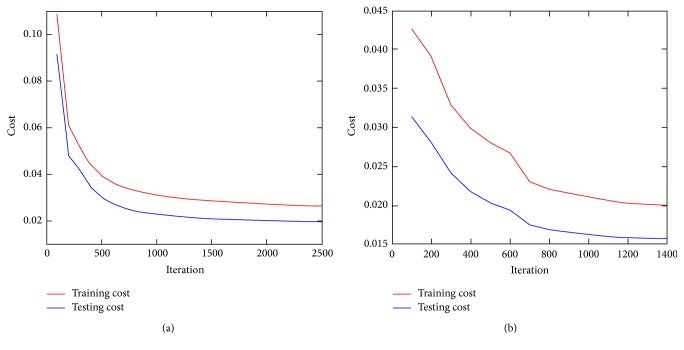
Convergence curves of different network architectures, W-Net (a) or V-Net (b).

Exemplary detection results of 3 slices of different body regions using deep learning methods are visualized in axial plane in Figure 5, where the false positive and false negative are marked out for in-depth comparison. Typically, false negatives occur when the lesion is too small while the contrast is not high enough to identify its presence. False positives are highly intensity driven, which considers the nonspecific high tracer uptake as lesion by mistake. The V-Net with CT or PET alone predicted lesions in low accuracy with lots of false negative and false positives. The V-Net with hybrid PET/CT data as input is capable of learning features from both modalities. It can generally prevent false positive prediction to a large extent. For W-Net, the obtained binary skeleton mask is forwarded to the second V-Net together with PET/CT volumes. Therefore, W-Net geometrically offers extra anatomical restrictions and reduces the probability of assigning wrong lesion labels, which further improves the detection performance compared to V-Net of the same input. The convergence curves for different networks are shown in Figure 6.

The experimental results of conventional machine learning methods (RF, *k*-NN, and SVM) are shown in Table 1. For the traditional methods, all of them achieve comparable good sensitivity and specificity. However, to the exact voxelwise discrimination of correct classes, random forest obtains a Dice score of 21.69%, *k*-NN gives a Dice score of 23.09%, and SVM shows a Dice score of 26.94%. The outperformance of SVM over random forest and *k*-NN indicates that SVM is more capable of providing a hyperplane in distinguishing nonlesion regions from lesion regions than the other two. For random forest, it might be explained as that bone lesions are in quite a small quantity compared to the rest of healthy parts of the whole body, which results in the data to be rather sparse and hinders its performance. This is exactly the advantage when applying sparse data to SVM. For *k*-NN, the reason behind it might be that the pure calculation of Euclidean distance as the features to predict the closeness of testing voxel and the training samples does not fit for bone lesions spread over the full body.

For the first time, this study proposed a deep learning method to automatically detect and segment the whole-body MM bone lesions on CXCR-4 PET/CT imaging. It can efficiently leverage the potential information inside multimodal imaging instead of extracting handcrafted features that are difficult to identify or reach an evaluation consensus. However, the current study is restricted by small number of patient data. Even though we tried to augment the number of training samples by generating patches, the performance of the deep learning methods is still hampered. Nevertheless, this explorative study demonstrated the potential of deep learning methods in combining multimodal information for lesion detection. The preliminary results support the further development of deep learning methods for whole-body lesion detection. With the evolution of CXCR-4 imaging and therapy in clinical practice, more and more subjects will be enrolled for the tests. The performance of deep learning is expected to be improved with the availability of more data volumes. Besides, we only focus on the detection of bone lesions of multiple myeloma in this proof-of-concept study and lesions outside the bone are not considered. This is not realistic for multiple myeloma patients with possible extramedullary lesions. The W-Net architecture can be naturally extended to the detection of lesions outside the bone by incorporating more labels of other tissue types and lesions. However, this needs sufficient data to make the training and test converge, which may be achieved with an increased number of subjects.

## 4. Conclusion

This study proposed the first computer-aided MM bone lesion detection approach on whole-body ^68^Ga-Pentixafor PET/CT imaging. It explored two deep learning architectures, that is, V-Net and W-Net, for lesion segmentation and detection. The deep learning methods can efficiently combine the information inside multimodal imaging and do not require the extraction of handcrafted features that are difficult to identify with regard to intermodality characteristics. We demonstrate the feasibility of deep learning methods by conducting realistic digital phantom study. Traditional machine learning methods were also compared to further confirm the advantage of deep learning approaches in handling lesion heterogeneities. The preliminary results based on limited number of data support the W-Net, which incorporates additional skeletal information as a kind of regularization for MM bone lesion detection. Increasing the amount of data may further enhance the performance of the proposed deep learning method. The trial of this study makes a step further towards developing an automated tool for the management of multiple myeloma disease.

## Figures and Tables

**Table 1:** Experimental results of phantom study using synthetic PET/CT using V-Net, random forest (RF), *k*-Nearest Neighbor (*k*-NN), and support vector machine (SVM).

Performance (%)	Sensitivity	Specificity	Precision	Dice
V-Net	89.71	99.68	88.82	89.26
RF with *n* = 20	99.16	89.49	12.18	21.69
kNN with *k* = 15	98.52	90.38	12.41	23.09
SVM with *C* = 0.5	98.76	92.15	15.60	26.94

**Table 2:** Experimental results of V-Nets and W-Net for MM bone lesion detection. Best results are indicated in italic.

Performance (%)	Sensitivity	Specificity	Precision	Dice
V-Net + CT	73.18	94.43	16.08	26.37
V-Net + PET	61.77	96.04	18.53	28.51
V-Net + PET/CT	71.06	99.51	68.00	69.49
*W-Net + PET/CT*	*73.50*	*99.59*	*72.46*	*72.98*
